# A survey of UK acute clinicians' knowledge of personal protective requirements for infectious diseases and chemical, biological, and radiological warfare agents

**DOI:** 10.1186/cc14166

**Published:** 2015-03-16

**Authors:** AR Bond, A Buckingham, J Schumacher

**Affiliations:** 1 Guy's and St Thomas' NHS Trust, London, UK; 2 St George's Healthcare NHS Trust, London, UK

## Introduction

We conducted a survey to assess clinicians' knowledge of personal protective equipment (PPE) requirements for infectious diseases and biochemical warfare agents. A safe level of PPE is essential when treating patients with highly infectious diseases or those contaminated with hazardous substances. The recent Ebola virus disease (EVD) outbreak in West Africa has highlighted that, although uncommon, contagious diseases with high mortality rates can be a threat to healthcare systems at local, national, and international levels [1]. Chemical, biological, radiological or nuclear (CBRN) contamination presents similar risks.

## Methods

A validated, hand-delivered, multiple-choice questionnaire [2] was used to assess intensive care, emergency medicine, and anesthetics specialist registrars' knowledge of respiratory and skin protection needed during a resuscitation scenario with advanced life support. Participants selected the PPE required for the biological hazards: EVD, severe acute respiratory syndrome (SARS), inhalational anthrax, plague and smallpox; and the biochemical hazards: sarin, hydrogen cyanide, phosgene and mustard gas (dichlordiethyl sulfide). Responses were compared with UK national recommendations and a previous survey in 2009 [2].

## Results

Ninety-eight clinicians (anesthetics *n *= 51, emergency medicine *n *= 21, intensive care medicine *n *= 26) completed surveys. The best knowledge (76% correct) was for SARS, with less knowledge for anthrax, plague, EVD, and smallpox (60%). We found limited knowledge for chemical warfare agents (20 to 30%). Sixty to 80% of all incorrect responses were over-rated. There was no difference in knowledge compared with previous published results [2].

## Conclusion

Despite national and regional training since previous surveys [2], the results indicate that further training on PPE is required for clinicians treating patients exposed to infectious diseases and CBRN agents, ideally in a simulation setting. Further research into whether the required levels of PPE are readily available to clinicians would be pertinent.
